# Multi-layer Scaffolds of Poly(caprolactone), Poly(glycerol sebacate) and Bioactive Glasses Manufactured by Combined 3D Printing and Electrospinning

**DOI:** 10.3390/nano10040626

**Published:** 2020-03-28

**Authors:** Adja B. R. Touré, Elisa Mele, Jamieson K. Christie

**Affiliations:** 1 Department of Materials, Loughborough University, Loughborough LE11 3TU, UK; e.mele2@lboro.ac.uk (E.M.); j.k.christie@lboro.ac.uk (J.K.C.); 2 Centre for Additive Manufacturing, Faculty of Engineering, Jubilee Campus, Nottingham University, Nottingham NG7 2RD, UK

**Keywords:** composite biomaterials, porous scaffolds, degradation

## Abstract

Three-dimensional (3D) printing has been combined with electrospinning to manufacture multi-layered polymer/glass scaffolds that possess multi-scale porosity, are mechanically robust, release bioactive compounds, degrade at a controlled rate and are biocompatible. Fibrous mats of poly (caprolactone) (PCL) and poly (glycerol sebacate) (PGS) have been directly electrospun on one side of 3D-printed grids of PCL-PGS blends containing bioactive glasses (BGs). The excellent adhesion between layers has resulted in composite scaffolds with a Young’s modulus of 240–310 MPa, higher than that of 3D-printed grids (125–280 MPa, without the electrospun layer). The scaffolds degraded in vitro by releasing PGS and BGs, reaching a weight loss of ~14% after 56 days of incubation. Although the hydrolysis of PGS resulted in the acidification of the buffer medium (to a pH of 5.3–5.4), the release of alkaline ions from the BGs balanced that out and brought the pH back to 6.0. Cytotoxicity tests performed on fibroblasts showed that the PCL-PGS-BGs constructs were biocompatible, with cell viability of above 125% at day 2. This study demonstrates the fabrication of systems with engineered properties by the synergy of diverse technologies and materials (organic and inorganic) for potential applications in tendon and ligament tissue engineering.

## 1. Introduction

In the field of tissue engineering, combinations of different materials and fabrication approaches are investigated to manufacture scaffolds that are able to satisfy more than one of the following requirements simultaneously [1,2,3]: be biocompatible; offer a biomimetic interface and multiscale porosity to promote cell attachment, migration and proliferation; mimic the mechanical response of the native tissue; and degrade, if required, at a controlled rate to support tissue regeneration. 

Recently, blends of poly(caprolactone) (PCL), a slow-degrading polyester, and poly(glycerol sebacate) (PGS), a fast-degrading polyester, have been processed by either electrospinning or 3D printing to create porous and biodegradable scaffolds with controlled mechanical properties for heart valve replacement [4,5,6], cardiac patches [7,8,9] and corneal tissue repair [10]. In one study, the degradation of PGS-PCL electrospun scaffolds (with a 2:1 PGS:PCL weight ratio) has been investigated in accelerated conditions in an alkaline medium (0.1 mM NaOH) and in vitro using valvular interstitial cells (VICs) [5]. Blending PGS with PCL led to a 16% mass loss of the scaffolds in 7 days, while fibrous mats of just PCL showed a 6% mass loss at the same timepoint. The PGS-PCL scaffolds stimulated VICs to secrete extracellular matrix (ECM) proteins (collagen I, laminin and fibronectin), which contributed to the mechanical properties of the cell-seeded scaffolds. A Young’s modulus of 9.3 MPa was recorded for PGS-PCL scaffolds seeded with VICs after 3 weeks of culture (7.8 MPa at day 0); while acellular scaffolds showed a two-fold decrease in elastic modulus under the same conditions. The enhanced secretion of ECM proteins and the control achieved over degradation and mechanical properties of the scaffolds indicate that the PGS-PCL electrospun mats can be used for heart valve tissue engineering.

In another work, PGS-PCL electrospun fibres have been functionalised with vascular endothelial growth factors (VEGFs) and tested as cardiac patches [7]. The PGS-PCL fibres (with a 2:1 PGS:PCL weight ratio) were characterised by a Young’s modulus of 8 MPa, which decreased to 1.4 MPa after 28 days of degradation in phosphate-buffered saline solution (PBS) at 37 °C, due to the breakdown of the ester bond and hydrolysis of PGS (resulting in 57% weight loss). VEGF was immobilised onto the scaffolds by 1-ethyl-3-(3-dimethylaminopropyl) carbodimide hydrochloride)/N-hydroxysuccinimide (EDC-NHS) chemistry and its release kinetics were investigated in water at 37 °C. The scaffolds showed a VEGF release of around 90% after 30 days and almost complete release after 50 days, possibly caused by the surface hydrolytic degradation of the fibres. The VEGF-functionalised mats promoted the adhesion and growth of mouse myoblast cell line C2C12 and rat cardiac progenitor cells (rCPCs), showing potential as tissue-engineered cardiac patches.

Aiming at improving cardiac remodelling after myocardial infarction, 3D printing of mixtures of PGS-PCL (with a PGS:PCL 9:1 weight ratio) and sacrificial sodium chloride particles has been performed [9]. Due to the high PGS concentration and 60% porosity, the scaffolds exhibited a Young’s modulus of 0.7 MPa and fast degradation (90% in 12 h) in highly concentrated PBS solution of lipase from *Thermomyces lanuginosus*. The scaffolds were implanted in adult male rats 2 days after left coronary ligation. Four weeks after implantation, the 3D-printed PGS-PCL scaffolds induced improvements in cardiac functions, decreased infarct size, and increased the thickness of the left ventricle wall and tissue remodelling. 

TO date, electrospinning and 3D printing of PCL-PGS blends have been demonstrated separately to obtain scaffolds with controlled properties. Here, we investigate, for the first time, PCL-PGS constructs consisting of electrospun mats deposited onto 3D-printed scaffolds. Bioactive glasses have been incorporated into the 3D printed polymer matrix to achieve better control over mechanical properties, degradation profile and biocompatibility. The composite PCL-PGS scaffolds formed of electrospun and 3D printed layers were characterised by a Young’s modulus in the range of 250–310 MPa, and experienced a weight loss of 10–15% after 56 days of incubation in a buffer solution. Hybrid fabrication strategies, like the combination of electrospinning and 3D printing, are discussed in the literature to introduce biomimetic features and multiscale porosity within additive manufactured scaffolds [11]. Here, the 3D printed layer provided mechanical support, maintained the shape integrity of the composite scaffolds and allowed the release of bioactive compounds. The electrospun layer was instead used to further engineer mechanical properties and porosity, and make biological inducible structures available to cells.

## 2. Materials and Methods 

### 2.1. Synthesis of PGS

PGS was synthesised following the procedure reported by Wang and collaborators [12]. A round-bottomed flask equipped with a N_2_ bubbler and a Dean–Stark trap was filled with an equimolar mixture of anhydrous glycerol (Sigma Aldrich, purity 99%, Gillingham, UK) and recrystallized sebacic acid (Sigma Aldrich, purity 99%). Bubbling N_2_ was passed through the mixture for 10 min before the compounds were heated up to 120 °C for 24 h. The pressure was then reduced from 1 torr to 40 mtorr over 5 h. The reaction mixture was kept at 40 mtorr and 120 °C for 48 h to crosslink the prepolymer.

### 2.2. Fabrication of 3D Printed Scaffolds

An extrusion printer 3D-Bioplotter Developer Series (EnvisionTec, Gladbeck, Germany) was used to produce three types of 3D printed scaffolds: PCL-PGS (hereafter referred to as 3D PCL-PGS), PCL-PGS containing 5 wt% of bioactive glasses (3D PCL-PGS-5BGs), and PCL-PGS containing 10 wt% of bioactive glasses (3D PCL-PGS-10BGs). PCL with a molecular weight of ~80,000 Da (Sigma Aldrich) was used. The bioactive glass (BG) microspheres (45S5, XL Tech science, Richland, WA, USA) were formed of 45.0 wt% SiO_2_, 24.5 wt% CaO, 24.5 wt% Na_2_O and 6 wt% P_2_O_5_. The BGs (0.25 and 0.50 g for 3D PCL-PGS-5BGs and 3D PCL-PGS-10BGs, respectively) were firstly dispersed in 50 μL of dimethyl sulfoxide (DMSO, Sigma Aldrich) and 10 mL of acetone (Sigma Aldrich). The dispersion was stirred for 1 h using a magnetic stirrer under vigorous agitation, before adding 2.5 g of PGS. Following the complete dissolution of PGS at 40 °C, 2.5 g of PCL were added, and the dispersion was left stirring at 40 °C for 12 h.

Barrels containing the solutions were individually loaded into the Bioplotter and dispensed through smooth-flow tapered tips (340 µm internal diameter) from 12.7 mm and extruded on to an aluminium build platform coated with an aluminium foil. Pressure and speed time for pre-flow and post-flow were adjusted and optimised to obtain the desirable inner structure for each composition. The printing process was conducted at 37 °C. The samples (square grids, area 4 × 4 cm^2^) consisted of layers formed of parallel strands placed at a 1.45 mm distance from one to another. Each layer was deposited perpendicular to the previous one, in order to form a grid.

For the fabrication of multi-layer constructs, the 3D-printed samples were left to dry on the aluminium foil and then used as a collector for the electrospinning process. PCL and PGS were mixed at 1:1 weight ratio and dissolved in a 7:3 (weight ratio) mixture of dichloromethane (Sigma Aldrich) and methanol (Sigma Aldrich) to obtain a 14 wt% polymer solution. A plastic syringe with a 21G needle (0.80 mm in diameter and 120 mm in length) was filled with the polymer solution, prepared, and connected to a syringe pump (New Era Pump System, NE-300, New York, NY, USA), working at a flow rate of 0.8 ml/h. The needle was connected to the positive electrode of a high-voltage power supply (S1500032-0, Linari Engineering s.r.l., Pisa PI, Itlay), generating a voltage of 8 kV, while the ground electrode was fixed to an aluminium collector (air gap distance of 15 cm). All experiments were conducted in normal environmental conditions. The PCL-PGS solution was electrospun only on one surface of the 3D-printed scaffolds. After production, the samples were stored at room temperature.

### 2.3. Characterisation Procedures

#### 2.3.1. Nuclear Magnetic Resonance

^1^H Nuclear Magnetic Resonance (NMR) spectra of PGS were recorded in commercial deuterated solvent on a JEOL ECS-400 spectrometer (JEOL (UK) Ltd., Herts, UK; ^1^H at 399.782 MHz, ^13^C at 100.525 MHz), a Bruker Advance Ultra-Shield 400 spectrometer (Bruker AXS Ltd., Coventry, UK; ^1^H at 400.134 MHz, ^13^C at 100.624 MHz) at 293 K. Chemical shifts were expressed as δ in parts per million (ppm) and were adjusted to the chemical shift of the residual NMR solvent resonance peak (CDCl_3_, ^1^H: δ = 7.26 ppm).

#### 2.3.2. Fourier Transform Infrared Spectroscopy

The infrared spectra of PGS were recorded on the neat compound using a Fourier Transform Infrared (FTIR) Spectrophotometer Shimadzu FTIR-8400S (Shimadzu Europa Gmbh, Duisburg, Germany), equipped with an Attenuated Total Reflection (ATR) diamond and irradiating between 7800 and 350 cm^−1^, at 64 scans and a resolution of 4 cm^−1^. The data were recorded through the software IR solution.

#### 2.3.3. X-ray Diffraction

The BG microspheres were dispersed in acetone and the suspension was placed onto a silicon substrate to produce a thin smear. The sample was analysed by a Bruker D8 Advance diffractometer (Bruker AXS Ltd.) in reflection geometry, Cu Kα radiation. The data were collected over the *2θ* range from 5° to 60° with a step size of 0.014° and a count time of 5.5 s per step. 

#### 2.3.4. Scanning Electron Microscopy

The morphology of BG microspheres, electrospun fibres and 3D printed scaffolds was investigated by Field Emission Gun Scanning Electron Microscopy (FEGSEM, LEO 1530VP, LEO Elektronenmikroskopie GmbH, Oberkochen, Germany). Prior to observation, the samples were stuck on aluminium stubs by carbon adhesive tapes and then coated using a palladium/gold sputter coater for 90 s (Emitech SC7640 Sputter Coater, Polaron, Laughton, UK) to produce a conductive surface. 

#### 2.3.5. Uniaxial Tensile Tests

The mechanical properties of the scaffolds were analysed by a single column tabletop testing system Instron 5944 at room temperature in air. The samples were cut in rectangular strips 20 mm wide and 40 mm long, and mounted with side action grip clamps with flat jaw faces. The average sample thickness was (205 ± 4) µm for the composition 3D-ES PCL-PGS, (240 ± 9) µm for 3D-ES PCL-PGS-5BGs and (280 ± 9) µm for 3D-ES PCL-PGS-10BGs. The rate of extension was set at 10 mm/min. Ten samples for each scaffold composition were analysed and the data are given in the text as average ± standard deviation. The load vs. extension graphs show the average curve within a shaded region, which indicates the values measured for all replicates. The elastic moduli of all samples were calculated within the linear region of the stress-strain curve between 0.001–0.005 strain.

#### 2.3.6. In vitro Degradation Tests

The degradation behaviour of the scaffolds was evaluated in PBS solution in static conditions at 37 °C for 3 months. PCL-PGS scaffolds without BGs were used as control. Changes in pH, water absorption and weight loss were recorded. Squared samples (1 cm^2^) were placed in 24-well plates with low evaporation lid in a humid atmosphere, and 2.5 mL of PBS was added to cover the samples completely. To determine initial dry weight, the samples were weighed with a 0.1 mg resolution balance (Sartoruis, CP3245). The plates were then incubated at 37 °C under humid conditions. For each timepoint, the weight loss of the samples and pH changes in the PBS solution were recorded (five measurements per time point). The weight loss was calculated by recording the mass of the samples before (*m_i_*) and after (*m_f_*) the degradation period: weight loss = (*m_f_* − *m_i_*)/*m_i_*. The samples were then washed with deionised water and left to dry overnight, before weighing again to determine the degraded dry weight (*m_d_*). The percentage water absorption of each sample was calculated as *(m_f_ − m_d_)/m_d_*.

The mechanical properties of 2 × 4 cm^2^ rectangular samples, incubated in PBS at 37 °C, were also analysed after one and two months of degradation.

#### 2.3.7. Biocompatibility Tests

3T3 cells (fibroblasts) were harvested and cultured from mice. After thawing, the 3T3 cells were grown in Alpha MEM Medium (BioWhittaker Reagents, Lonza Inc, Manchester, UK) that was complemented with 10% foetal bovine serum (Sigma Aldrich), 1% antibiotic and antimycotic (100 µg/ml penicillin, 100 mg/ml streptomycin, and 0.25 µg/ml amphotericin B; Sigma Aldrich) and 1% of L-Glutamine (Sigma Aldrich). Cell cultures were maintained in 75 cm^2^ flasks (Costar, Corning Inc., Corning, NY, USA) in standard culture conditions of 37 °C and 5% CO_2_. Cells were passaged every 2 days after harvesting them by trypsinization using 0.05% Trypsin-EDTA solution (Gibco, Fisher Scientific, Loughborough, UK) at 80–90% culture confluence and further sub-cultivated into culture flasks. 3T3 cells were counted using Trypan blue (Sigma Aldrich) and a hemocytometer. The cells were seeded in 96-well flat-bottomed plates using a pipette. A total of 10,000 cells were deposited in 36 wells of a 96-well plate.

The scaffolds (with an area of 1 cm^2^) were sterilised using ethanol, following a procedure reported in the literature [8,13,14]. Three samples for each type of scaffold were tested. The scaffolds were covered with 2.5 mL of media and placed in an incubator under standard culture conditions of 37 °C and 5% CO_2_. A total of 2.5 mL of medium without scaffold or cells was incubated alongside the others. After 1, 3 and 7 days, 0.2 mL of the medium covering the scaffolds was taken using a pipette and placed on top of the cells in the 96-well plates. Negative control samples (cells) were incubated in the culture medium Alpha MEM; some of them were incubated alongside the scaffolds. Positive control samples (cells) were treated with Industrial Methylated Spirit (IMS) just 20 min before testing cell viability. Following this step, the cells were incubated for 24 h and viability was measured using the PrestoBlue Cell Viability Reagent Protocol and assessed using fluorescence measurements. All conditions were tested in triplicate wells.

After being incubated for 24 h, culture supernatant was slowly aspirated using a pipette linked to an aspirator. 0.1 ml of 1/10 PrestoBlue in medium solution was deposited in each well plate containing cells and in three wells without cells to measure the background fluorescence of the PrestoBlue. The well plate was covered with aluminium foil and placed in an incubator for 30 min. Fluorescence/viability was assessed using a plate reader. During the reading, the 96-well plate was agitated for 5 s, and measurements of fluorescence were done at 560 nm excitation and 590 nm emission. Measurements corresponding to the negative control were considered as 100% cell viability. Viability values for the other samples were normalised to this value.

## 3. Results and Discussion

**Figure 1:**
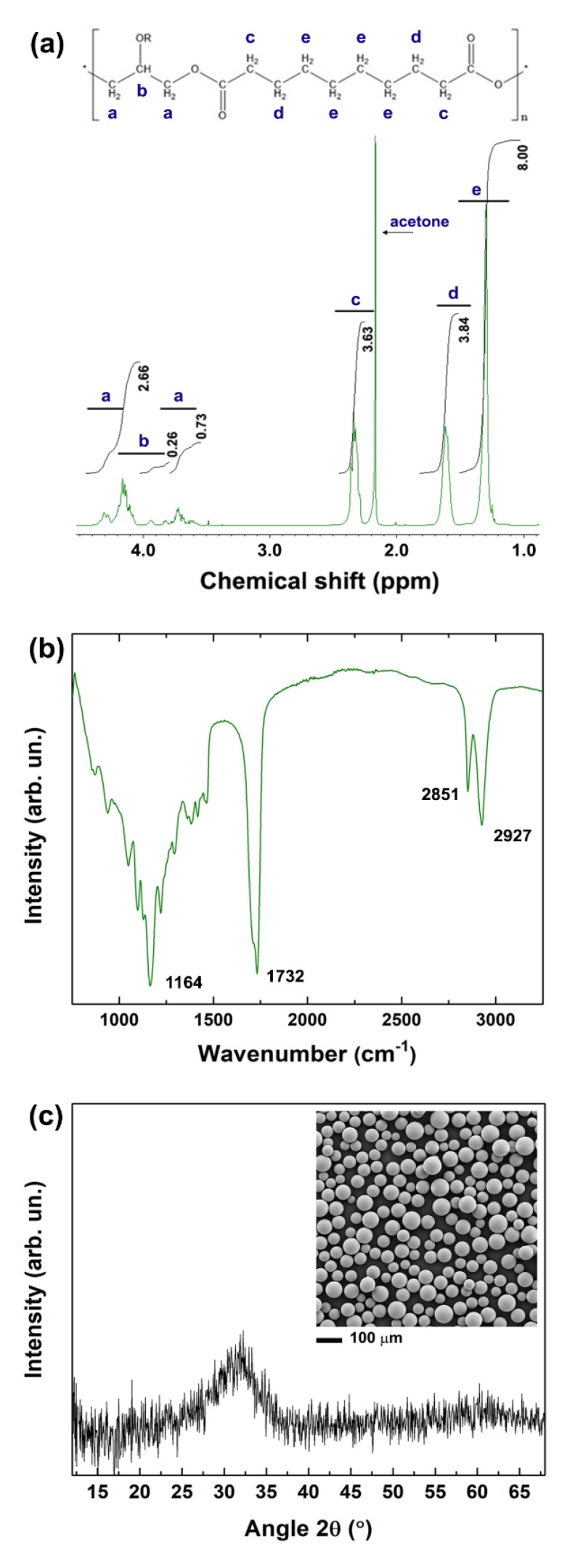
(**a**) 1H NMR spectrum of poly (glycerol sebacate) (PGS) in deuterated acetone. The typical chemical structure of PGS is shown at the top of the graph. (**b**) FTIR spectrum of PGS. The characteristic peaks are indicated in the graph. (**c**) XRD pattern of the 45S5 bioactive glass microspheres and their morphology analysed by SEM (inset).

Here, as demonstrated by NMR and FTIR analyses, PGS was successfully synthesised using a two-step procedure: polycondensation of glycerol and sebacic acid, followed by thermal crosslinking [12]. The ^1^H NMR spectrum in Figure 1a shows the methylene peaks of sebacic acid at 1.30, 1.62 and 2.35 ppm, and the peaks of glycerol between 4.05 and 4.35 ppm [15]. The signature bands of PGS are identifiable also in the FTIR spectrum (Figure 1b): the peaks at 2927 and 2851 cm^−1^ are attributed to alkene (–CH_2_) groups; the intense peaks at 1732 and 1164 cm^−1^ are due to C=O and C–O stretching, respectively, and confirm the formation of ester bonds [16,17,18].

### 3.1. Fabrication of Multi-layer PCL-PGS-BGs Scaffolds

After synthesis, PGS was blended with PCL (in a 1:1 PCL:PGS weight ratio) to produce multi-layer scaffolds consisting of a 3D-printed grid coated with a mat of electrospun fibres. Bioactive glass microspheres with an average diameter of (60 ± 25) μm (inset of Figure 1c) were incorporated into the 3D printed layer to achieve control over mechanical properties and degradation behaviour. The amorphous nature of the BG microspheres used was confirmed by the XRD pattern in Figure 1c, showing a broad band at diffraction angles between 25° and 35°, due to the short-range order of the silicate structure [19,20]. 

**Figure 2:**
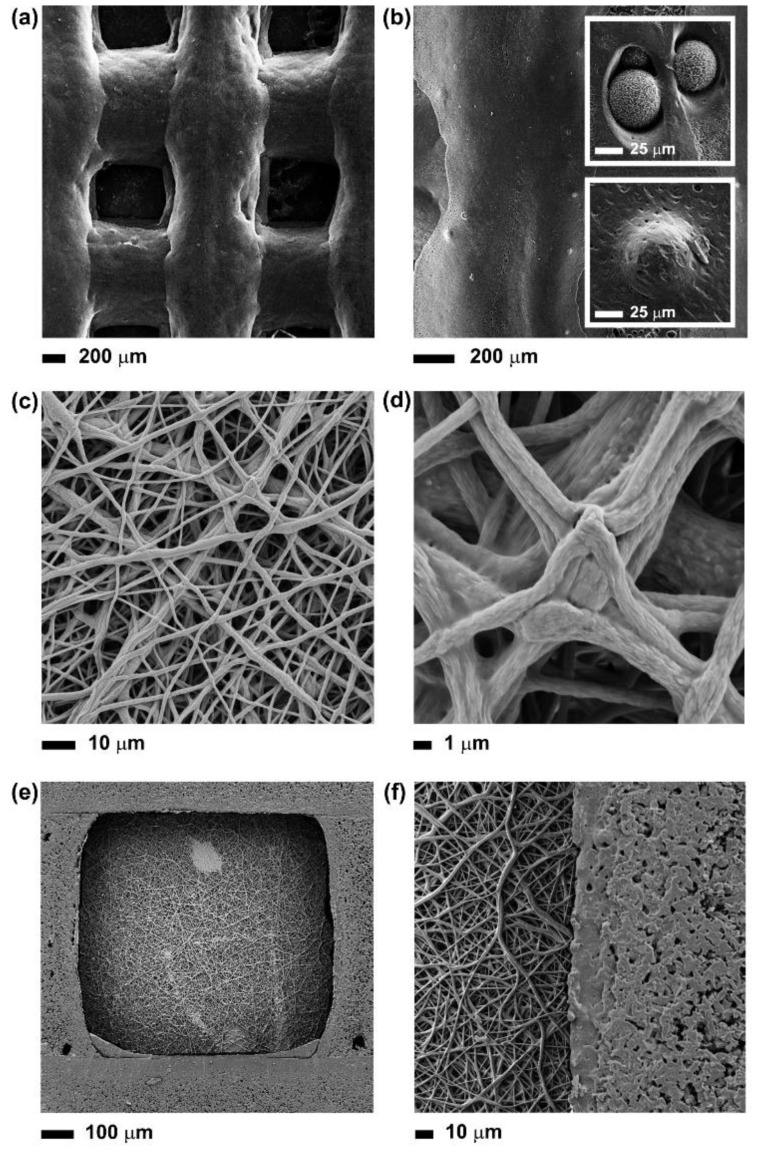
SEM images at different magnifications of: (**a**) and (**b**) the 3D-printed scaffolds, showing also the BG microspheres exposed onto the surface or embedded into the polymer matrix (insets); (**c**) and (**d**) the surface of the composite scaffold covered with a layer of electrospun PCL-PGS mats, pointing out the fusion between fibres; (**e**) and (**f**) the surface of the composite scaffold without the layer of electrospun fibres.

The 3D-printed grids were characterised by interconnected pores poly-dispersed in three dimensions, due to the presence of solvent residues after layer deposition (Figure 2a,b). On one hand, the solvent facilitated the 3D printing process by reducing the viscosity of the PCL-PGS solution, promoting the dispersion of the BG microspheres, and therefore avoiding clogging the nozzle during printing. On the other hand, the low viscosity of the polymer blend determined the deformation and fusion of layers. The first layer was less affected by the deformation, while the second and the subsequent layers were squeezed and radially diffused, with the consequent formation of geometrically irregular pores. Deviations from perfect square pores have been observed in previous studies on the extrusion printing of reticular structures of soft materials, such as hydrogels and elastomers [21,22]. The fusion between adjacent PCL-PGS printed layers, due to solvent traces and low melting temperature of PGS (broad range between −20 and 40 °C) [16], was advantageous to create monolithic constructs with improved mechanical stability (Section 3.2). As shown in Figure 2b and relative insets, the BG microspheres were predominantly uniformly distributed within the whole volume of the scaffold, even if small aggregates were also observed. The BGs were exposed on the surface of the 3D-printed scaffolds but also embedded into the polymer matrix. The exposed BG microspheres and the surface micro-porosity of the scaffolds, caused by solvent evaporation, contributed to the erosion of the systems during degradation (as discussed in Section 3.3).

One side of the 3D printed structures (the final 3D printed layer) was coated with a mat of PCL-PGS electrospun fibres. In order to achieve a strong adhesion between layers, the electrospinning process was conducted directly onto the 3D printed constructs. The resulting network was free from defects or beads, and formed of bundles of fibres (Figure 2c,d). the fusion between adjacent PCL-PGS fibres has been reported in the literature and attributed to the slow evaporation of the solvent [13]. The low voltage used to extrude the fibres (8 kV instead of 15–18 kV reported in previous studies on 1:1 PCL:PGS solutions) [4,23] limited jet elongation and fibre stretching, likely slowing down solvent evaporation during fibre collection [24,25]. The fibres reached the collector (the 3D-printed scaffold) before complete solidification; the trapped solvent continued to diffuse out and determined coalescence at the fibre–fibre junctions and along the fibre length. Therefore, membranes consisting of interconnected fibre layers were created. The presence of solvent traces in the fibres was essential to promote adhesion between the electrospun mat and the 3D-printed structure, and ensure uniform coverage of the pores of the 3D grid (Figure 2e,f). The adhesion was tested by keeping the multi-layer PCL-PGS scaffolds in water at 37 °C under agitation for one week. No signs of detachment of the electrospun mat from the 3D printed grid were observed.

In a previous study on biphasic systems formed of electrospun PCL fibres and 3D PCL/beta tri-calcium phosphate (β-TCP) scaffolds, direct electrospinning onto the 3D struts was not successful in obtaining strong adhesion, due to the absence of solvent in the fibres [26]. It was instead necessary to partially melt one surface of the 3D scaffold and press it into the electrospun mat, with consequent changes in the morphology and porosity of this latter layer. In a recent work, PCL electrospun sheets have been treated with oxygen plasma, before being inserted in between cross-linked 3D-printed alginate layers [27]. The plasma treatment made the PCL fibres hydrophilic and prevented layer delamination. Our composite PCL-PGS scaffolds were instead ready to be used after electrospinning, without the need for post-processing procedures.

### 3.2. Characterisation of the Tensile Response of the PCL-PGS-BGs Scaffolds

**Figure 3:**
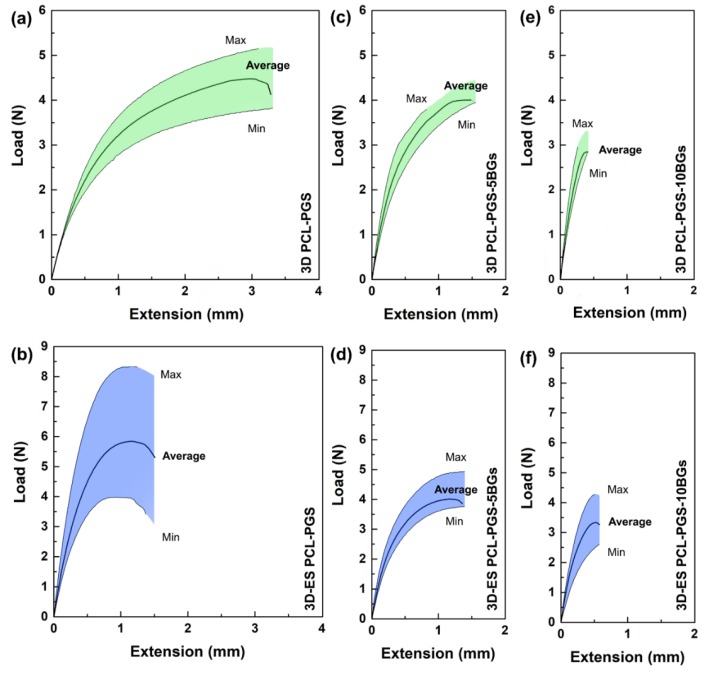
Load vs. extension curves of: 3D printed PCL-PGS, without (**a**) and with (**b**) the electrospun PCL-PGS mat; 3D printed PCL-PGS with 5 wt% of BGs, without (**c**) and with (**d**) the electrospun PCL-PGS mat; 3D printed PCL-PGS with 10 wt% of BGs, without (**e**) and with (**f**) the electrospun PCL-PGS mat. For each sample, the average curve is shown within a shaded region, which indicates the range of load values measured for ten replicates.

The mechanical properties of the composite scaffolds were investigated by uniaxial tensile tests. The 3D-printed PCL-PGS grids sustained an average maximum load of 4.5 N in tension and a maximum extension of 3.5 mm (Figure 3a). The addition of the electrospun layer resulted in scaffolds (referred to as 3D-ES PCL-PGS) that bore a maximum load of 6.0 N and extended up to 1.5 mm (Figure 3b). The electrospun fibres acted as a reinforcement for the assembled patches due to the excellent fibre–fibre bonding and strong interfacial adhesion between the electrospun layer and the 3D-printed one. Similar mechanical behaviour, even though for a different scaffold architecture, has been recorded for three-layer systems made of porous PGS tubes wrapped with PCL electrospun fibres and reinforced by PCL rings (tensile load at break of around 5.5 N), where PGS significantly contributed to the elastomeric response [28]. The use of electrospun sheets to increase tensile strength in composites has been described in the literature for microfibre-based netting materials [29,30,31]. 

The incorporation of 5wt% of bioactive glass microspheres in the PCL-PGS matrix gave a significant reduction in the sample extension before failure, with no statistically significant changes in the maximum load (Figure 3c). Similar values of load and extension at the breaking point were recorded after the deposition of the fibrous layer (Figure 3d). By increasing the BGs concentration to 10 wt%, drops in both maximum load and extension were recorded for 3D PCL-PGS–10BGs (Figure 3e) and 3D-ES PCL-PGS–10BGs (Figure 3f).

**Figure 4:**
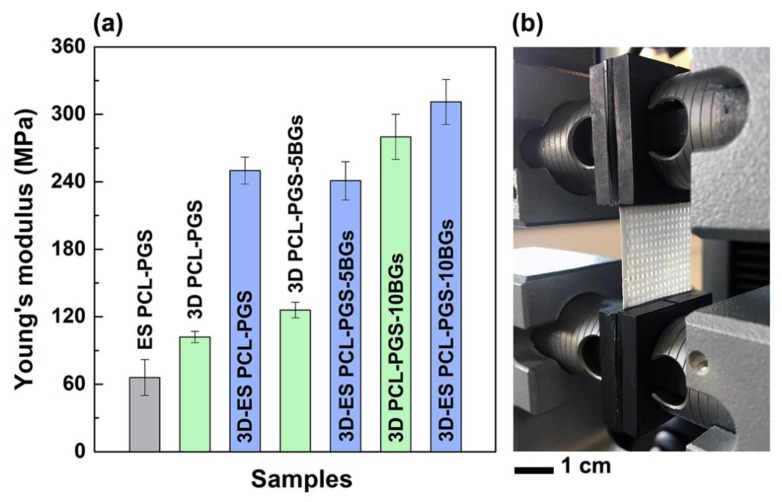
Values of Young’s modulus for the different scaffold types (**a**). The photograph (**b**) of one composite scaffold during the tensile test shows the 3D printed grid covered by the electrospun mat.

The analysis of the Young’s modulus (*E*) better clarifies the role of the electrospun mat and BGs concentration on the mechanical properties of the scaffolds (Figure 4). The electrospun mats (ES PCL-PGS) exhibited a Young’s modulus of (66 ± 16) MPa, which is higher than values reported in the literature for electrospun 1:1 PCL:PGS blends (in the range of 3.6–11.0 MPa) [4,13]. The cross-linking of PGS and fusion between fibres and electrospun layers are likely to be the reasons for the high values measured. It has been reported that the Young’s modulus of PCL fibrous mats can be increased by welding procedures, such as vapour treatment and thermal annealing [32,33]. These methods induce interfibre bonding that limits fibre movement and slipping during stretching, with a consequent increase in mat resistance to deformation [34]. 

When the fibres were deposited onto the 3D printed PCL-PGS scaffolds, a significant rise in the Young’s modulus of the composite scaffolds was observed: from (102 ± 5) MPa for 3D PCL-PGS samples to (250 ± 12) MPa for 3D-ES PCL-PGS ones. The increase in stiffness can be associated with the excellent adhesion between the 3D-printed layer and the electrospun one, and makes the electrospun/3D-printed samples of potential interest as engineered scaffolds for stiff human tissues, such as tendons and ligaments. The effect of the electrospun fibres on the mechanical properties of the scaffolds was evident also for samples containing BG microspheres. 3D-printed scaffolds with 5 and 10 wt% of BGs had *E* values of (126 ± 7) MPa and (280 ± 20) MPa, respectively; the deposition of the electrospun mats determined an increase in Young’s modulus up to (241 ± 17) MPa for 3D-ES PCL-PGS-5BGs and (311 ± 20) MPa for 3D-ES PCL-PGS-10BGs. A comparison between composite 3D-ES samples shows that 5 wt% of BGs had no significant impact on the stiffness, differently from 10 wt% BGs that induced a 30% increase in the Young’s modulus. Similar values of elastic modulus in tension have been reported for polymer-BGs scaffolds for bone tissue engineering, including: 60μm-thick PCL films containing 50 wt% of 45S5 bio-glasses (~200 MPa) [35], electrospun mats (80 μm thick) of polyvinyl alcohol and 45S5 BGs (~250 MPa) [36], and polydimethylsiloxane-BGs-PCL monoliths (230–330 MPa) [37].

### 3.3. Analysis of the Degradation Behaviour of the PCL-PGS-BGs Scaffolds

**Figure 5:**
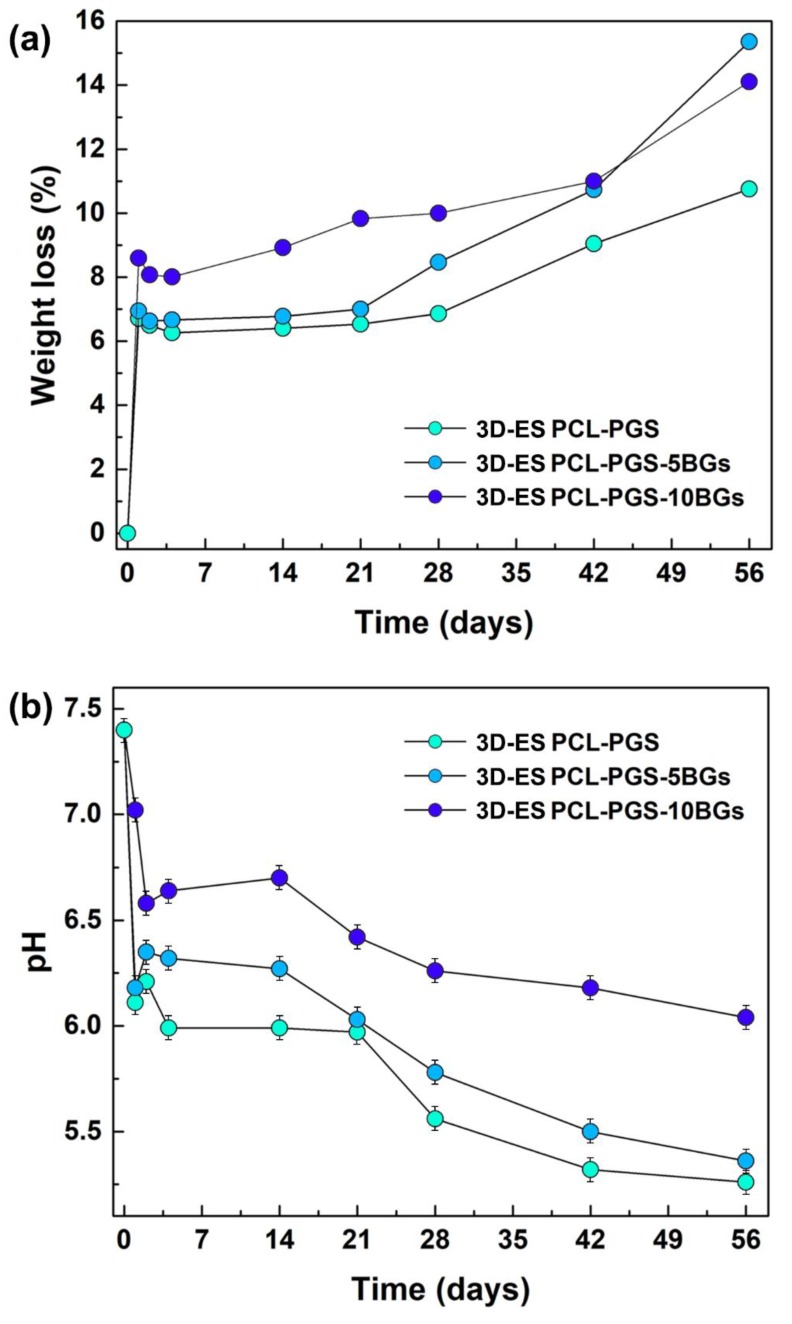
In vitro degradation of the composite scaffolds with and without bioactive glass microparticles: (**a**) percentage of weight loss and (**b**) pH changes at different timepoints. The data are represented as average ± standard deviation for five repeats (the size of the symbol includes the error bar).

The degradation of the composite scaffolds was studied in PBS at 37 °C and pH 7.4 to reproduce normal physiological conditions. Weight loss of the samples (Figure 5a) and pH changes in the buffer solution (Figure 5b) were recorded over a period of 3 months. The scaffolds without BGs and with 5 wt% BGs showed a similar degradation profile with a weight loss of 6.5% (3D-ES PCL-PGS) and 7.0% (3D-ES PCL-PGS-5BGs) after 21 days; while, in the same time frame, the 3D-ES PCL-PGS-10BGs samples exhibited a 9.8% weight loss. The degradation was initiated by the hydrolysis of PGS and dissolution of the BGs microparticles [38,39,40,41,42]. The release of unreacted carboxylic acid groups of PGS and those formed by the cleavage of PGS ester linkages caused acidification of the PBS medium [13,23]: pH 6.0 after 21 days. The release of alkaline ions from the scaffolds with the lowest concentration of BGs (5 wt%) had a limited effect on balancing out the pH drop (pH 6.0); while 10 wt% BGs maintained pH to values higher than 6.5 for the first 14 days. The weight loss continued to increase over time for all scaffolds, reaching 10.7% for 3D-ES PCL-PGS, 15.4% for 3D-ES PCL-PGS-5BGs and 14.1% for 3D-ES PCL-PGS-10BGs, after 56 days of incubation in PBS. This corresponded to a decrease in the pH of the PBS medium to 5.3–5.4 for 3D-ES PCL-PGS and 3D-ES PCL-PGS-5BGs, and 6.0 for 3D-ES PCL-PGS-10BGs.

**Figure 6:**
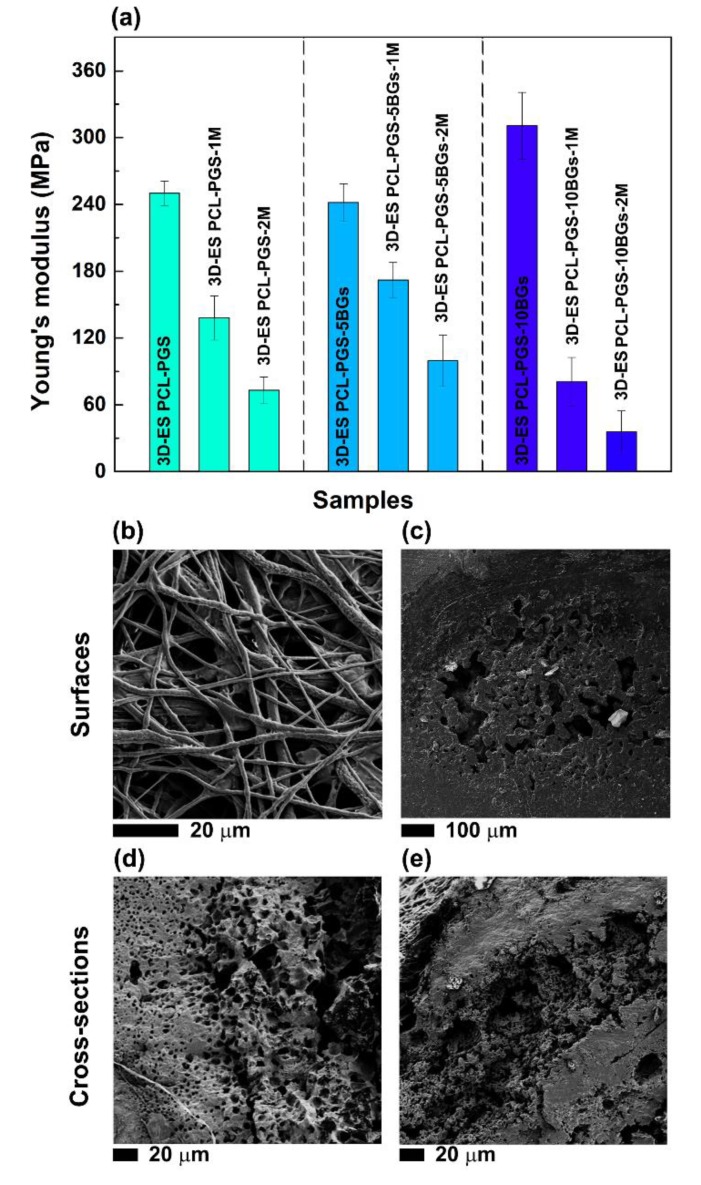
(**a**) Values of Young’s modulus of the composite scaffolds after one month (3D-ES PCL-PGS-1M, 3D-ES PCL-PGS-5BGs-1M, 3D-ES PCL-PGS-10BGs-1M) and two months (3D-ES PCL-PGS-2M, 3D-ES PCL-PGS-5BGs-2M, 3D-ES PCL-PGS-10BGs-2M) of incubation in PBS at 37 °C. SEM images, after 2 months degradation, of the surface (**b**) of the electrospun layer and (**c**) 3D-printed one; (**d**) and (**e**) the cross-section of the 3D-printed layer.

The weight loss experienced by scaffolds containing BGs indicates that both PGS and bioactive glass microparticles were released, as was also confirmed by the pH changes in the PBS medium, particularly for samples with 10 wt% BGs. As a consequence, a deterioration in the mechanical properties and morphology of the scaffolds was observed. As shown in Figure 6a, a significant decrease in the Young’s modulus was recorded for all scaffold types after one and two months of degradation. Samples with 10 wt% of BGs exhibited the lowest value of Young’s modulus, (36 ± 15) MPa, corresponding to a more than 8-fold decrease compared to the non-degraded scaffolds. SEM investigations of the electrospun layer revealed that non-fibrous regions (thin polymer films) started to emerge in between fibres and the roughness of the fibres surface increased (Figure 6b), likely due to the erosion of PGS. Pores and defects were instead detected on the surface (Figure 6c) and within the cross-section (Figure 6d,e) of the 3D-printed layer, caused by PGS and BGs release, which are ultimately responsible for the failure of the scaffolds under tension.

### 3.4. Biocompatibility Tests

**Figure 7:**
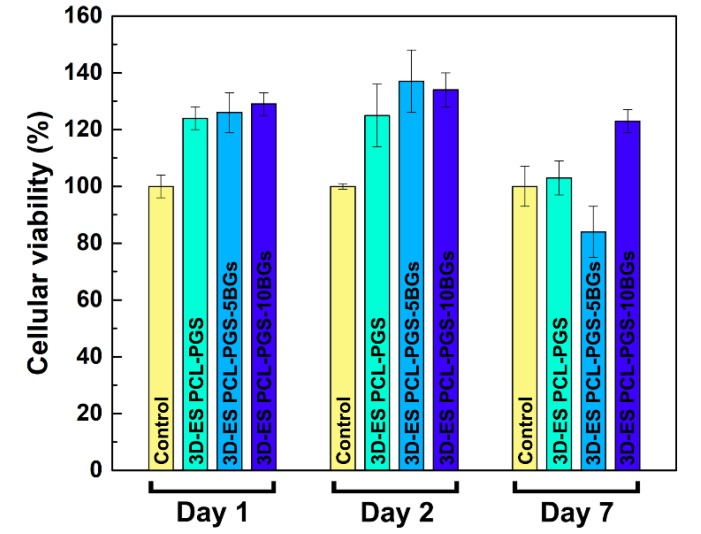
Percentage of 3T3 cell viability for 3D-ES PCL-PGS scaffolds without and with bioactive glasses for three times points (1, 2 and 7 days).

The viability of 3T3 cells exposed to the PCL-PGS extracts is shown in Figure 7. All three material compositions exhibited cell viability results above 80% for all timepoints, indicating satisfactory biocompatibility. High survival rates (higher than the control sample) were recorded for all scaffolds: 3D-ES PCL-PGS (124 ± 4%), 3D-ES PCL-PGS-5BGs (126 ± 7%) and 3D-ES PCL-PGS-10BGs (129 ± 4%) at day 1. The highest viability was measured at day 2 with values of 125% ± 11%, 137% ± 11% and 134% ± 6% for 3D-ES PCL-PGS, 3D-ES PCL-PGS-5BGs and 3D-ES PCL-PGS-10BGs, respectively. A decrease in cell survival was observed for all scaffold types at day 7, particularly for 3D-ES PCL-PGS-5BGs (84% ± 9%). The results of the biocompatibility tests can be linked to the degradation of PGS, the release profile of BGs and corresponding pH changes (as shown in Figure 5). Although PGS degradation could lead to an acidic environment deleterious for cells, the release of the alkaline BGs helped to counteract the low pH and ensured a suitable environment for cell proliferation. Viability results demonstrated the positive effect of the addition of BGs in the 3D constructs since most cell survival rates were above that of the reference sample without BGs (3D-ES PCL-PGS).

## 4. Conclusions

Through this work, the design, fabrication and characterisation of scaffolds meeting various requirements for tissue engineering have been demonstrated. Electrospinning and 3D printing have been combined to manufacture biocompatible patches of PCL-PGS blends containing bioactive glasses. The scaffolds exhibited a multiscale three-dimensional porosity that is known to be beneficial for cell growth and infiltration, and the transport of nutrients and metabolic waste (not demonstrated in this work). In addition, the use of PGS and bioactive glasses allowed control over degradation and mechanical properties. The strategy used for the fabrication of the hybrid scaffolds has proved effective in achieving excellent adhesion between the 3D-printed layer, which provided mechanical support to the whole structure, and the electrospun mat, which acted as a biomimetic layer. The presence of the PCL-PGS electrospun network enhanced the stiffness of the scaffolds and gave a 2.5-fold increase of the Young’s modulus. The addition of the bioactive glasses, although it affected the mechanical response of the scaffolds only slightly (a 30% increase in elastic modulus for samples with 10 wt% BGs), impacted the material degradation in vitro and balanced the acidic character of PGS. After 3 months of in vitro degradation tests, the pH of the PBS medium, where the scaffolds were incubated, dropped to 5.3 (from 7.4) when no BGs were used; while it only reached values of 6.0 for scaffolds containing 10 wt% BGs. This had a positive effect on the growth of fibroblasts, whose viability was sustained longer in vitro for scaffolds with 10 wt% BGs. The composite scaffolds can find potential applications in tendon and ligament tissue engineering, since they meet the mechanical requirements of native tissues (elastic modulus in the range of 100–300 MPa) [43], in agreement with previous studies [44]. For these specific tissue engineering applications, scaffolds with anisotropic characteristics can be manufactured using the fabrication method discussed in this work.
